# 4′-Fluorouridine is an oral antiviral that blocks respiratory syncytial virus and SARS-CoV-2 replication

**DOI:** 10.1126/science.abj5508

**Published:** 2021-12-02

**Authors:** Julien Sourimant, Carolin M. Lieber, Megha Aggarwal, Robert M. Cox, Josef D. Wolf, Jeong-Joong Yoon, Mart Toots, Chengin Ye, Zachary Sticher, Alexander A. Kolykhalov, Luis Martinez-Sobrido, Gregory R. Bluemling, Michael G. Natchus, George R. Painter, Richard K. Plemper

**Affiliations:** ^1^ Center for Translational Antiviral Research, Georgia State University, Atlanta, GA 30303, USA.; ^2^ Texas Biomedical Research Institute, San Antonio, TX 78227, USA.; ^3^ Emory Institute for Drug Development, Emory University, Atlanta, GA 30322, USA.; ^4^ Drug Innovation Ventures at Emory (DRIVE), Atlanta, GA 30322, USA.; ^5^ Department of Pharmacology, Emory University School of Medicine, Atlanta, GA 30322, USA.; ^6^ Department of Pediatrics, Emory University School of Medicine, Atlanta, GA 30322, USA.

## Abstract

The COVID-19 pandemic has underscored the critical need for broad-spectrum therapeutics against respiratory viruses. Respiratory syncytial virus (RSV) is a major threat to pediatric patients and the elderly. We describe 4′-fluorouridine (4′-FlU, EIDD-2749), a ribonucleoside analog that inhibits RSV, related RNA viruses, and SARS-CoV-2 with high selectivity index in cells and human airway epithelia organoids. Polymerase inhibition within in vitro RdRP assays established for RSV and SARS-CoV-2 revealed transcriptional stalling after incorporation. Once-daily oral treatment was highly efficacious at 5 mg/kg in RSV-infected mice or 20 mg/kg in ferrets infected with different SARS-CoV-2 variants-of-concern, initiated 24 or 12 hours after infection, respectively. These properties define 4′-FlU as a broad-spectrum candidate for the treatment of RSV, SARS-CoV-2, and related RNA virus infections.

**Figure:**
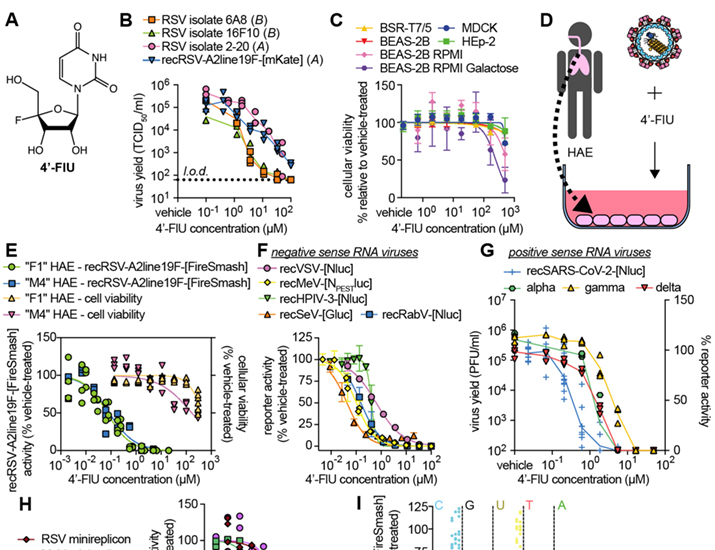

The COVID-19 experience has highlighted the need for orally-bioavailable broad-spectrum antivirals that could be quickly deployed against newly emerging viral pathogens. Remdesivir—a direct-acting broad-spectrum antiviral—is still the only small molecule therapeutic approved for use against SARS-CoV-2 infection in the United States, but it requires intravenous administration. The ensuing restriction to hospitalized patients compromises its clinical effect as treatment is initiated too late in the infection cycle (*1*). We have demonstrated efficacy of orally available EIDD-2801 (molnupiravir) against influenza viruses in human organoid models and ferrets (*2*), and subsequent animal and human data showed that antiviral efficacy of molnupiravir extends to SARS-CoV-2 in vivo (*3*, *4*). Molnupiravir acts by inducing lethal viral mutagenesis after incorporation into viral genomic RNA of influenza viruses (*2*) and betacoronaviruses (*5*). The drug was recently approved in the United Kingdom and is currently considered for emergency use authorization against COVID-19 in the United States. However, even with this accelerated development timeline, molnupiravir only became available to patients nearly two years into the pandemic. To have a substantial effect on a mounting pandemic, an antiviral must be approved for human use before a new pathogen emerges, making the case for the development of broad-spectrum antivirals.

We have identified RSV disease as a viable primary indication for a candidate broad-spectrum antiviral, on the basis of the unmet major health threat imposed by RSV and well-established protocols for clinical trials of anti-RSV therapeutics. RSV infections are responsible for over 58,000 hospitalizations of children below 5 years of age in the United States annually, and approximately 177,000 hospitalizations of adults above the age of 65 (*6*–*9*). Despite this major health and economic burden, no therapeutics have been licensed specifically for treatment of RSV disease (*10*). Anti-RSV drug discovery efforts have increasingly focused on inhibiting the viral RNA-dependent RNA polymerase (RdRP) complex (*11*). The core polymerase machinery comprises the large (L) polymerase protein, its obligatory cofactor, the phosphoprotein (P), and the encapsidated negative-sense RNA genome (*11*). Allosteric inhibitors of RSV L have potent activity as seen, for instance, with the experimental drug candidates AVG-233 (*12*) and inhaled PC786 (*13*).

In search of a drug that is active against RSV and SARS-CoV-2, is orally available, and acts through a distinct mechanism of activity (MOA) from molnupiravir, we explored 4′-fluorine substitutions in a series of analogs of the molnupiravir parent molecule *N*^4^-hydroxycytidine (NHC) (*14*). The focus on 4′-fluorine ribose substitutions was motivated by the small atomic radius and strong stereo-electronic effect of fluorine that can influence backbone conformation flexibility, which may lead to improved selectivity indices, increased lipophilicity, and greater metabolic stability (*15*). A synthetic intermediate in the approach to 4′-fluoro-*N*^4^-hydroxycytidine (compound 5 in fig. S1) was deprotected to provide 4′-FIU (Fig. 1A), which emerged as broadly active antiviral when biotested.

**Fig. 1.**
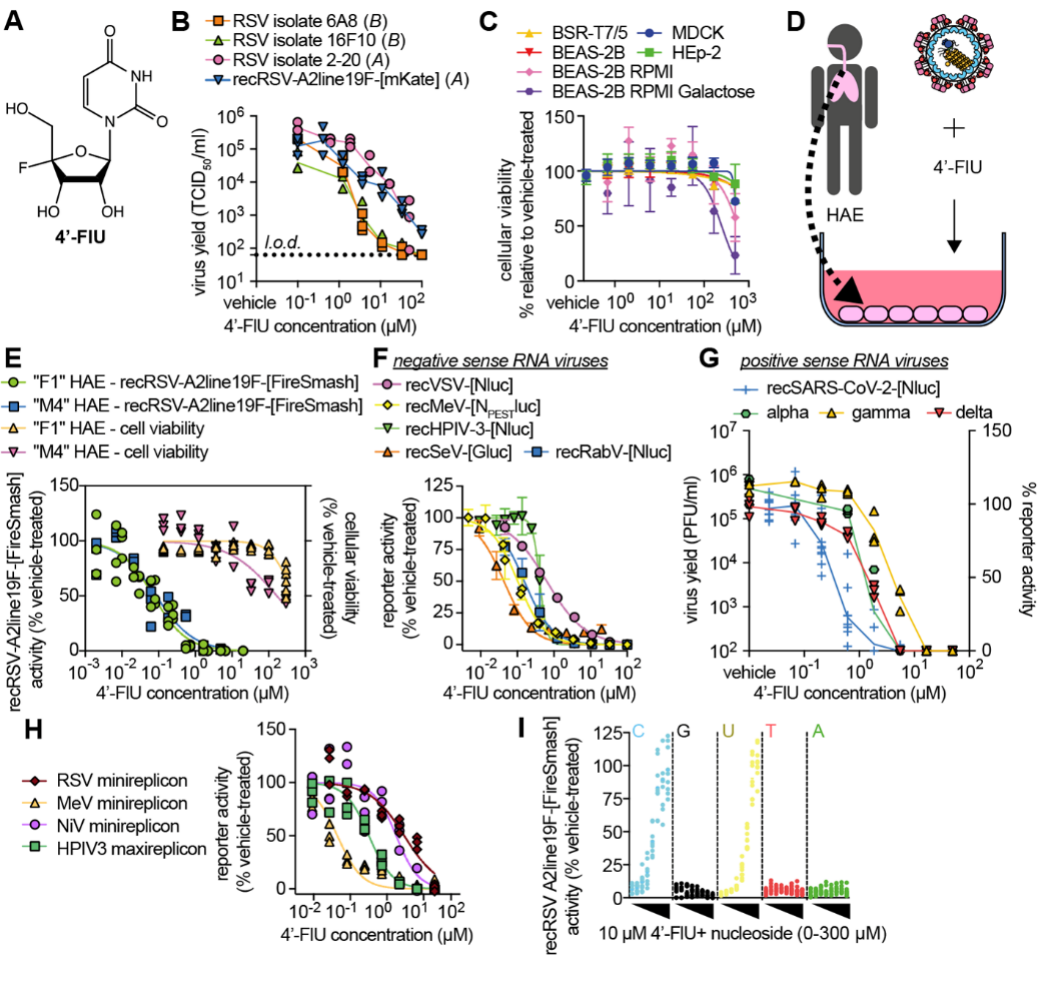
4′-FlU is a potent broad-spectrum antiviral. (**A**) Chemical structure of 4′-FlU. (**B**) Virus yield reduction of RSV clinical isolates 6A8, 16F10, 2-20, and recombinant recRSV-A2line19F-[mKate] [(A) or (B) antigenic subgroup]. (**C**) HEp-2, MDCK, BHK-T7, and BEAS-2B cell lines were assayed for reduction in cell metabolism by 4′-FlU. (**D **and** E**) recRSV-A2line19F-[FireSMASh] dose response inhibition and cytotoxicity assay with human airway epithelial (HAE) cells (D) from two donors in the presence of indicated 4′-FlU concentrations (E). (**F**) Dose-response inhibition of a panel of recombinant mononegaviruses by 4′-FlU. (**G**) Dose-response inhibition of recSARS-CoV-2-[Nluc] and virus yield reduction of alpha, gamma, and delta VoC isolates by 4′-FlU. (**H**) Dose-response inhibition of transiently expressed polymerase complexes from mononegaviruses MeV, RSV, NiV, or HPIV-3 by 4′-FlU (**I**) recRSV-A2line19F-[FireSMASh]-infected cells were treated with 10 μM of 4′-FlU and serial dilutions of exogenous nucleotides in extracellular media. Viral activity was determined by reporter activity. Symbols represent independent repeats (B), (E), (G), (H), and (I) or mean with standard deviation (C) and (F), and lines represent means. *n* ≥ 3, EC_50_s and CC_50_s are reported in tables S1 and S2, and all source data are provided in data S2.

## 4′-FlU is a broad-spectrum mononegavirus inhibitor with high SI

Following the approach of using RSV disease as a primary indication to advance a novel candidate broad-spectrum antiviral, we first assessed activity of 4′-FlU against a recombinant RSV A2-line19F (recRSV A2-L19F) (*16*) and clinical RSV isolates on immortalized HEp-2 cells. The compound showed potent dose-dependent activity against all RSV strains tested, returning half-maximal effective concentrations (EC_50_ values) ranging from 0.61 to 1.2 μM (Fig. 1B and table S1). This cell culture potency was on par with the previously reported anti-RSV activity of NHC (fig. S2). Global metabolic activity of established human and animal cell lines (HEp-2, MDCK, BHK-T7, and BEAS-2B) exposed to up to 500 μM of 4′-FlU remained unaltered, indicating that the antiviral effect is as a result of cytotoxicity (Fig. 1C and table S2). When glucose was replaced with galactose as a carbohydrate source to link cell metabolic activity strictly to mitochondrial oxidation (*17*), we determined a half-maximal cytotoxic concentration (CC_50_) of 4′-FlU of 250 μM (Fig. 1C and table S2).

When tested on disease-relevant primary human airway epithelial cells (HAE) derived from two different donors (Fig. 1D), 4′-FlU showed ≥17-fold increased anti-RSV potency relative to that on HEp-2 cells, but unchanged low cytotoxicity (CC_50_ 169 μM) (Fig. 1E), resulting in a high selectivity index (SI = EC_50_/CC_50_) of ≥1877. Consistent with these findings, quantitative immunocytochemistry on HAE cells confirmed that 4′-FlU reduced steady-state levels of nuclear-(SDH-A; IC_50_ 272.8 μM) and mitochondrial-(COX-I; IC_50_ 146.8 μM) encoded proteins only at high concentrations (fig. S3).

We next explored the 4′-FlU indication spectrum. We assessed a panel of negative-sense RNA viruses of the paramyxovirus and rhabdovirus families, including measles virus (MeV), human parainfluenza virus type 3 (HPIV3), Sendai virus (SeV), vesicular stomatitis virus (VSV), and rabies virus (RabV) that, like RSV, belong to the mononegavirus order, and found that the compound demonstrated submicromolar active concentrations (Fig. 1F and table S1). Testing a representative of phylogenetically distant positive-sense RNA viruses, the betacoronavirus SARS-CoV-2 was also sensitive to 4′-FlU, with EC_50_ values ranging from 0.2 to 0.6 μM against isolates of different lineages (Fig. 1G and table S1).

At initial mechanistic characterization, 4′-FlU inhibited RSV and paramyxovirus RdRP complex activity in cell-based minireplicon systems (Fig. 1H and table S1). The RdRP activity of Nipah virus (NiV)—a highly pathogenic zoonotic paramyxovirus with pandemic potential (*18*)—was also efficiently inhibited by 4′-FlU in an NiV minireplicon reporter assay. The antiviral effect of 4′-FlU was dose-dependently reversed by addition of an excess of exogenous pyrimidines (cytidine and uridine) but not purines to the cultured cells, which is consistent with competitive inhibition of RdRP activity (*2*, *19*) (Fig. 1I).

## Incorporation of 4′-FlU by RSV and SARS-CoV-2 RdRP causes sequence-modulated transcriptional stalling

**Fig. 2.**
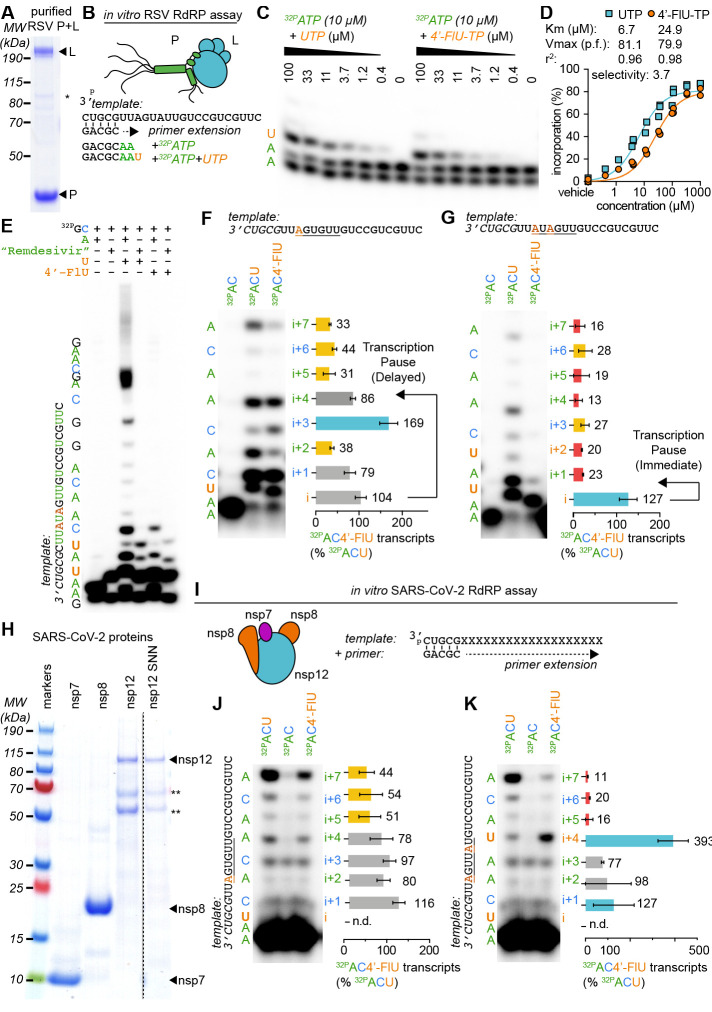
4′-FlU induces a delayed stalling of RSV and SARS-CoV-2 RdRP. (**A**) SDS-PAGE with Coomassie blue staining of recombinant RSV RdRP complexes (L and P proteins). (**B**) Schematics of the primer extension assay. (**C**) Urea-PAGE fractionation of RNA transcripts produced through primer extension by the RSV RdRP in the presence of the indicated nucleotides. (*n *= 3). (**D**) Kinetic analysis of autoradiographs from (C). Nonlinear regression with the Michaelis-Menten model. Km and Vmax with 95% confidence intervals (CIs) and goodness of fit (r^2^) are indicated. (**E **to** G**) Urea-PAGE fractionation of RNA transcripts produced by RSV RdRP in the presence of the indicated templates and nucleotides. “Remdesivir” denotes the addition of the remdesivir active metabolite GS-443902, a well-characterized “delayed chain terminator”. 4′-FlU-TP bands in (F) to (G) were normalized to the corresponding band after UTP incorporation. Bars represent mean and error bars represent standard deviation (*n *= 3). (**H**) Purified recombinant SARS-CoV-2 RdRP complexes (nsp7, 8, and 12 proteins) “nsp12 SNN” denotes a catalytically inactive mutant. (**I **to** K**) Urea-PAGE fractionation of RNA transcripts produced by SARS-CoV-2 RdRP in the presence of the indicated templates and nucleotides. Stars denote cellular contaminants. Uncropped autoradiograph replicates are provided in data S1.

To characterize the molecular MOA of 4′-FlU, we purified recombinant RSV L and P proteins expressed in insect cells (Fig. 2A) and determined performance of the bioactive 5′-triphosphate form of 4′-FlU (4′-FlU-TP) within in vitro primer extension assays (*20*) (Fig. 2B). In the presence of radio-labeled ATP and an increasing amount of UTP, RSV RdRP complexes elongated the primer until reaching a G in third position on the template strand, and continued further upon addition of CTP (Fig. 2C) (fig. S4 and data S1). Replacing UTP with 4′-FlU-TP resulted in efficient primer extension up to the third nucleotide, confirming that RSV RdRP recognizes and incorporates 4′-FlU in place of UTP (Fig. 2C). Incorporation kinetics (*21*) showed only a moderate reduction in substrate affinity for 4′-FlU-TP compared with UTP (Fig. 2D). Further addition of CTP to the reaction mix resulted in limited elongation rather than the expected full-length product, which suggested delayed polymerase stalling by incorporated 4′-FlU (fig. S4 and data S1). Direct side-by-side comparison with GS-443902—the active metabolite of remdesivir and a “delayed polymerase stalling” inhibitor well-characterized for SARS-CoV-2—along with RSV and other RNA viruses (*21*, *22*), corroborated this antiviral effect of 4′FlU-TP (Fig. 2E and data S1).

When a modified template coded for incorporation of only a single UTP (Fig. 2F and data S1), primers elongated preferentially to position *i*+3 after 4′-FlU-TP, whereas the efficiency of full elongation was strongly reduced compared with extension in the presence of UTP. However, repositioning the incorporation site further downstream in the template triggered immediate polymerase stalling at position *i* (fig. S5), indicating template sequence dependence of the inhibitory effect. Transcription stalling at *i* or *i*+3 were also observed after multiple 4′-FlU incorporations: an AxAxxx template (Fig. 2G) and direct tandem incorporations through an AAxxAx template (fig. S5) caused stalling at position *i*, whereas increasing spacer length between the incorporated uridines shifted preferential stalling to *i*+3 (fig. S5). This variable delayed polymerase stalling within one to four nucleotides of the incorporation site was equally prominent when we examined de novo initiation of RNA synthesis at the promoter with a synthetic native RSV promoter sequence rather than extension of primer-template pairs (fig. S6).

Purification of a core SARS-CoV-2 polymerase complex [nonstructural proteins (nsp) 7, 8, and 12] from bacterial cell lysates (*23*, *24*) (Fig. 2H) and assessment of RdRP bioactivity in equivalent primer-extension in vitro polymerase assays (Fig. 2I) again demonstrated incorporation of 4′-FlU-TP in place of UTP by the coronavirus RdRP (Fig. 2J), but no sign of immediate polymerase stalling. However, SARS-CoV-2 polymerase stalling was triggered by multiple incorporations of 4′-FlU-TP, and was particularly prominent when a second incorporation of 4′-FlU-TP occurred at the *i*+4 position (Fig. 2K and fig. S7). Primer extension was blocked when the nsp12 subunit was omitted or an nsp12 variant carrying mutations in the catalytic site was used, confirming specificity of the reaction (fig. S7 and data S1).

## 4′-FlU is rapidly anabolized, metabolically stable, and potently antiviral in disease-relevant well-differentiated HAE cultures

**Fig. 3.**
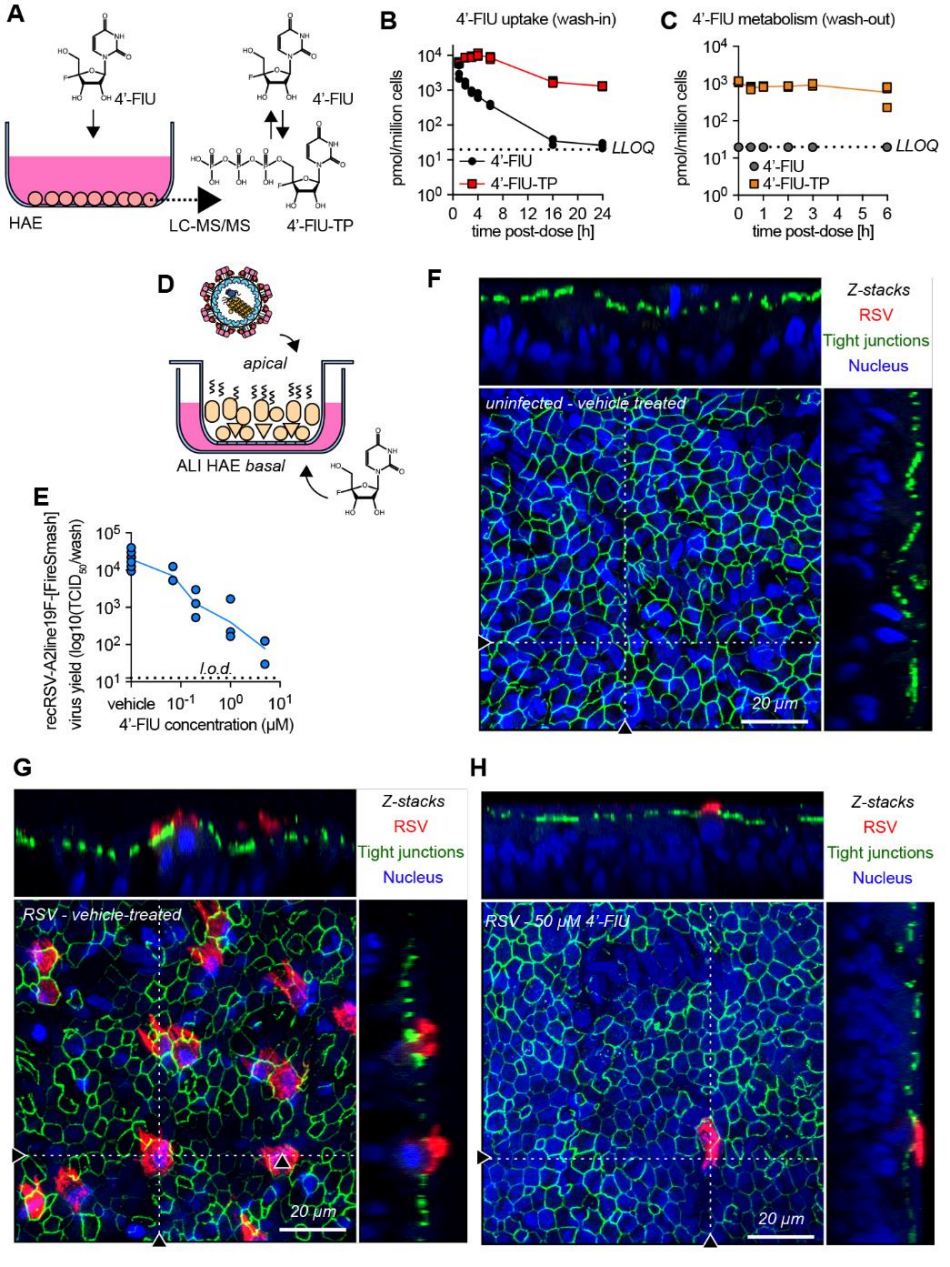
4′-FlU is efficiently anabolized in HAE cells and is efficacious in human airway epithelium organoids. (**A **to** C**) 4′-FlU cellular uptake and metabolism in “F1” HAE cells quantified by mass spectrometry (A). Intracellular concentration of 4′-FlU(-TP) after exposure to 20 μM 4′-FlU for 0, 1, 2, 3, 4, 6, 16, and 24 hours (B), or 24-hour incubation followed by removal of the compound for 0, 0.5, 1, 2, 3, and 6 hours before quantification (C) (*n *= 3). The low limit of quantitation (LLOQ) for 4′FlU (19.83 pmol/10^6^ cells) is indicated by the dashed line. (**D**) HAE cells were matured at air-liquid interface (ALI). (**E**) Virus yield reduction of recRSV-A2line19F-[FireSMASh] was shed from the apical side in ALI HAE after incubation with serial dilutions of 4′-FlU on the basal side (*n *= 3). (**F **to** H**) Confocal microscopy of ALI HAE cells infected with recRSV-A2line19F-[FireSMASh], at 5 days after infection. RSV infected cells, tight junctions, and nuclei were stained with anti-RSV, anti-ZO-1, and Hoechst 34580. z-stacks of 30 × 1 μm slices with 63 × oil objective. Dotted lines, x-z and y-z stacks; scale bar, 20 μm. In all panels, symbols represent independent biological repeats and lines represent means.

Quantitation of 4′-FlU and its anabolites in primary HAE cells (Fig. 3A) demonstrated rapid intracellular accumulation of 4′-FlU, reaching a level of 3.42 nmol/million cells in the first hour of exposure (Fig. 3B). Anabolism to bioactive 4′-FlU-TP was efficient, resulting in concentrations of 10.38 nmol/million cells at peak (4 hours after exposure start) and 1.31 nmol/million cells at plateau (24 hours). The anabolite was metabolically stable, remaining present in sustained concentrations of approximately 1 nmol/million cells over a 6-hour monitoring period, corresponding to an extrapolated half-life of 9.7 hours (Fig. 3C).

To explore efficacy in a disease-relevant human tissue model, we cultured the HAEs at the air-liquid interface, inducing the formation of a well-differentiated 3D airway epithelium that included ciliated and mucus-producing cells (*25*) (Fig. 3D). Adding 4′-FlU to the basolateral chamber of the transwells after apical infection of the epithelium with RSV potently reduced apical virus shedding with an EC_50_ of 55 nM (Fig. 3E). Overall titer reduction spanned nearly four orders of magnitude, ranging from 3.86×10^4^ TCID_50_ in control cells to 78.18 TCID_50_ at 5 μM basolateral 4′-FlU, approaching the level of detection.

Confocal microscopy validated formation of a pseudostratified organization of the epithelium with tight junctions in the airway epithelium tissue model (Fig. 3F), visualized efficient RSV replication in vehicle-treated tissue models (Fig. 3G), and confirmed near-sterilizing antiviral efficacy in the presence of 50 μM basolateral 4′-FlU (Fig. 3H and figs. S8 and S9). Under the latter conditions, positive staining for RSV antigen was rarely detected.

## 4′-FlU is orally efficacious in a therapeutic dosing regimen in a small-animal model of RSV infection

**Fig. 4.**
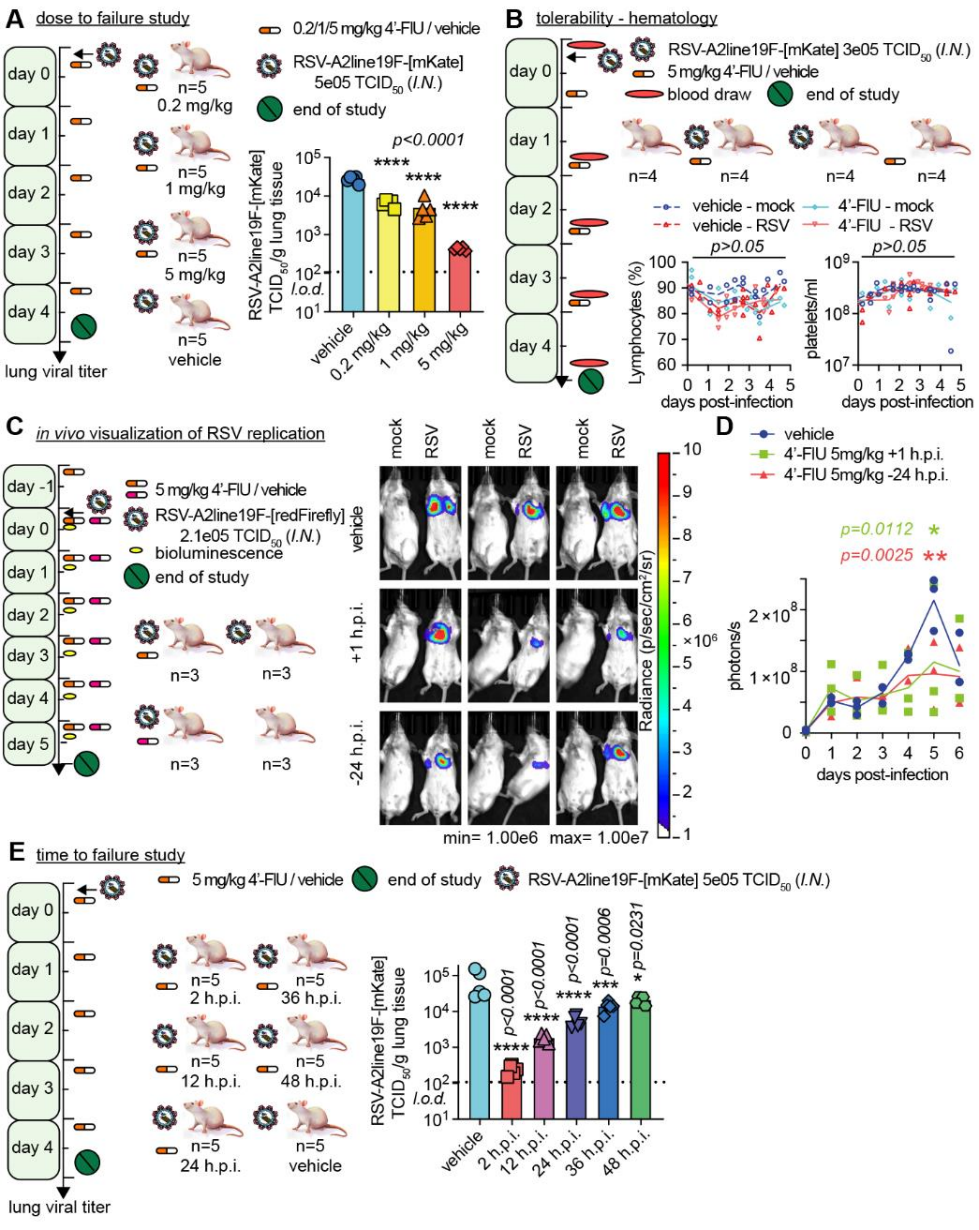
Therapeutic oral efficacy of 4′-FlU in the RSV mouse model. (**A**) Balb/cJ mice were inoculated with recRSV-A2line19F-[mKate] and treated as indicated. At 4.5 days after infection, viral lung titers were determined with TCID_50_ titration (*n *= 5). (**B**) Balb/cJ mice were inoculated with recRSV-A2line19F-[mKate] or mock-infected, and treated as indicated. Blood samples were collected before infection and at 1.5, 2.5, 3.5, and 4.5 days after infection, and lymphocyte proportions with platelets/ml are represented over time (*n *= 4). (**C**) Balb/cJ mice were inoculated with recRSV-A2line19F-[redFirefly] and treated as indicated. In vivo luciferase activity was measured daily. (**D**) Total photon flux from mice lungs from (C) over time (*n *= 3). (**E**) Balb/cJ mice were inoculated with recRSV-A2line19F-[mKate] and treated as indicated. At 4.5 days after infection, viral lung titers were determined with TCID_50_ titration (*n *= 5). In all panels, symbols represent individual values, and bars or lines represent means. One-way ordinary analysis of variance (ANOVA) with Tukey’s post hoc multiple comparisons (B) and (I) or two-way ANOVA with Dunnett’s post hoc multiple comparison (C) and (G). h.p.i., hours post-infection.

To test 4′-FlU efficacy in vivo, we employed the mouse model of RSV infection (supplementary text), challenging animals with recRSV-A2-L19F, which efficiently replicates in mice (*16*). In a dose-to-failure study, we infected BALB/cJ mice intranasally and initiated once-daily oral treatment two hours after infection at 0.2, 1, or 5 mg 4′-FlU/kg body weight. Treatment at all dose levels resulted in a statistically significant reduction in lung virus load compared with vehicle-treated animals (Fig. 4A). The antiviral effect was dose dependent and approached nearly two orders of magnitude at the 5 mg/kg dose. Consistent with high metabolic stability in HAEs, a twice-daily dosing regimen did not significantly enhance efficacy (fig. S10). Becuase animal appearance, body weight, temperature (fig. S11), and relative lymphocyte and platelet counts (Fig. 4B and fig. S12) were unchanged in the 5 mg/kg group compared with vehicle-treated animals, we selected this dose for further studies.

For a longitudinal assessment of therapeutic benefit, we employed an in vivo imaging system (IVIS) with a red-shifted luciferase (*26*) expressing a RSV reporter virus generated for this study. This assay allows a noninvasive spatial appreciation of intrahost viral dissemination. Daily imaging (Fig. 4C and fig. S13) revealed considerable reduction of bioluminescence intensity in lungs of 4′-FlU-treated animals at day 5 post-infection, corresponding to peak viral replication, independent of whether treatment was initiated 24 hours before or 1 hour after infection (Fig. 4D). This IVIS profile is consistent with reduced viral replication and ameliorated viral pneumonia in treated animals.

To probe the therapeutic window of 4′-FlU, we initiated treatment at 2, 12, 24, 36, and 48 hours after infection. All treatment groups showed a statistically significant reduction of lung virus burden compared with vehicle-treated animals, but effect size was dependent on the time of treatment initiation (Fig. 4E and fig. S14). On the basis of our experience with therapeutic intervention with related respiratory RNA viruses that cause lethal disease (*25*), we require a reduction of lung virus load of at least one order of magnitude. With this constraint, the therapeutic window of 4′-FlU extended to 24 hours after infection in mice.

## 4′-FlU is effective against SARS-CoV-2 in HAE and the ferret model

**Fig. 5.**
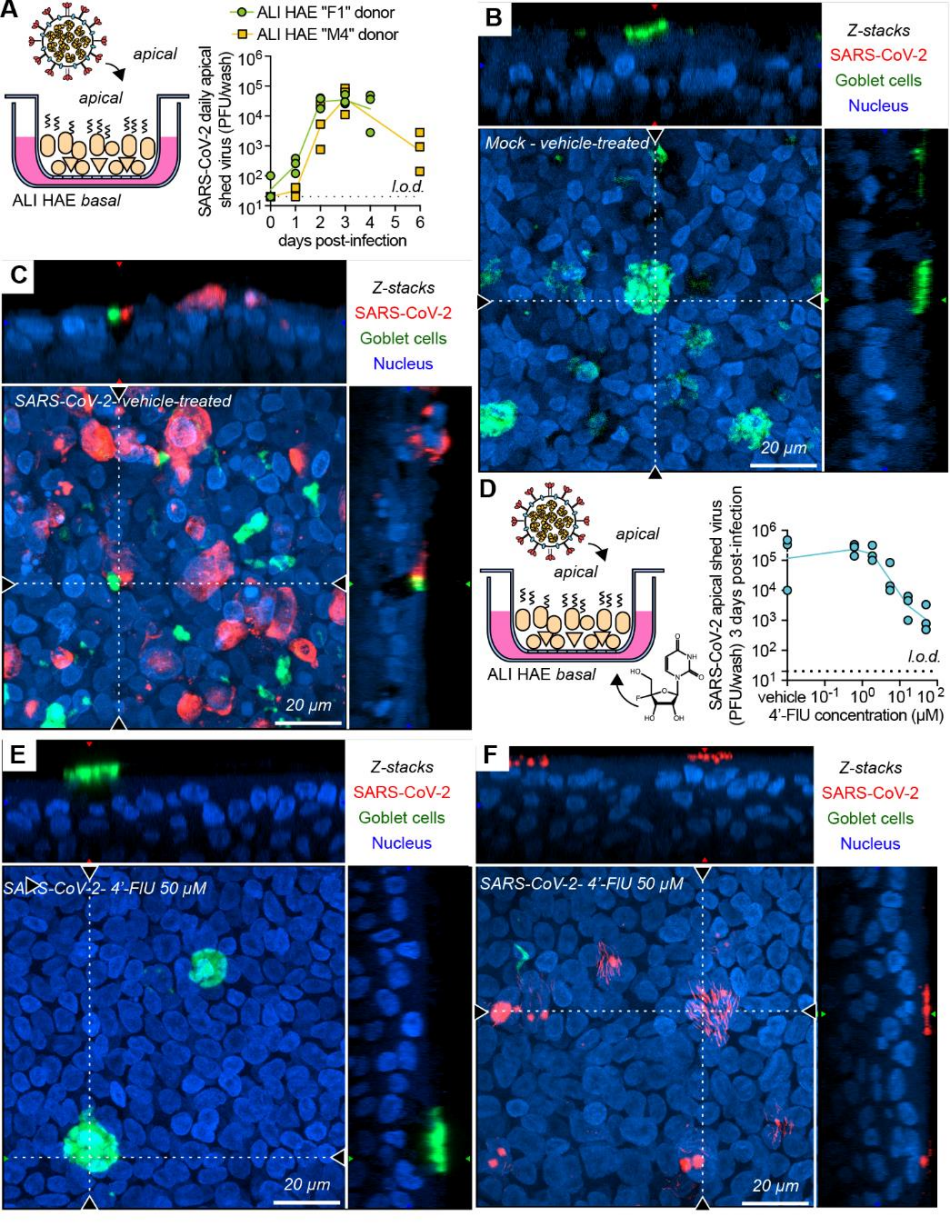
Efficacy of 4′-FlU against SARS-CoV-2 replication in HAE organoids. (**A**) Multicycle growth curve of SARS-CoV-2 WA1 isolate on ALI HAE from two donors. Shed virus was harvested daily and titered by plaque assay (*n *= 3). (**B **and** C**) Confocal microscopy of ALI HAE cells from “F1” donor mock-infected (B) or infected (C) with SARS-CoV-2 WA1 isolate, at 3 days post-infection. SARS-CoV-2 infected cells, goblet cells, and nuclei were stained with anti-SARS-CoV-2 N immunostaining, anti-MUC5AC immunostaining, and Hoechst 34580, pseudo-colored in red, green, and blue, respectively. z-stacks of 35 μm slices (1 μm thick) with 63× objective with oil immersion. Dotted lines represent the location of x-z and y-z stacks; scale bar, 20 μm. In all panels, symbols represent independent biological repeats and lines represent means. (**D**) Virus yield reduction of SARS-CoV-2 WA1 clinical isolate shed from the apical side in ALI HAE after incubation with serial dilutions of 4′-FlU on the basal side (*n *= 3). (**E** and** F**) Confocal microscopy of ALI HAE cells infected with SARS-CoV-2 WA1 isolate, and treated with 50 μM 4′-FlU 3 days after infection. Rare ciliated cells positive for N are represented in (F).

To test activity against SARS-CoV-2 in the human airway organoids, we first confirmed that the WA1 isolate replicated efficiently in HAEs of all donors tested (Fig. 5, A to C, and fig. S15). Treatment of infected organoids with basolateral 4′-FlU dose-dependently reduced apical virus shedding, albeit with a limited maximal effect size of approximately two orders of magnitude at 50 μM (Fig. 5D). Confocal microscopy revealed that the epithelium was largely devoid of SARS-CoV-2 nucleocapsid protein under these conditions (Fig. 5E), with only sporadic staining detectable in a small subset of ciliated cells (Fig. 5F and fig. S15).

**Fig. 6.**
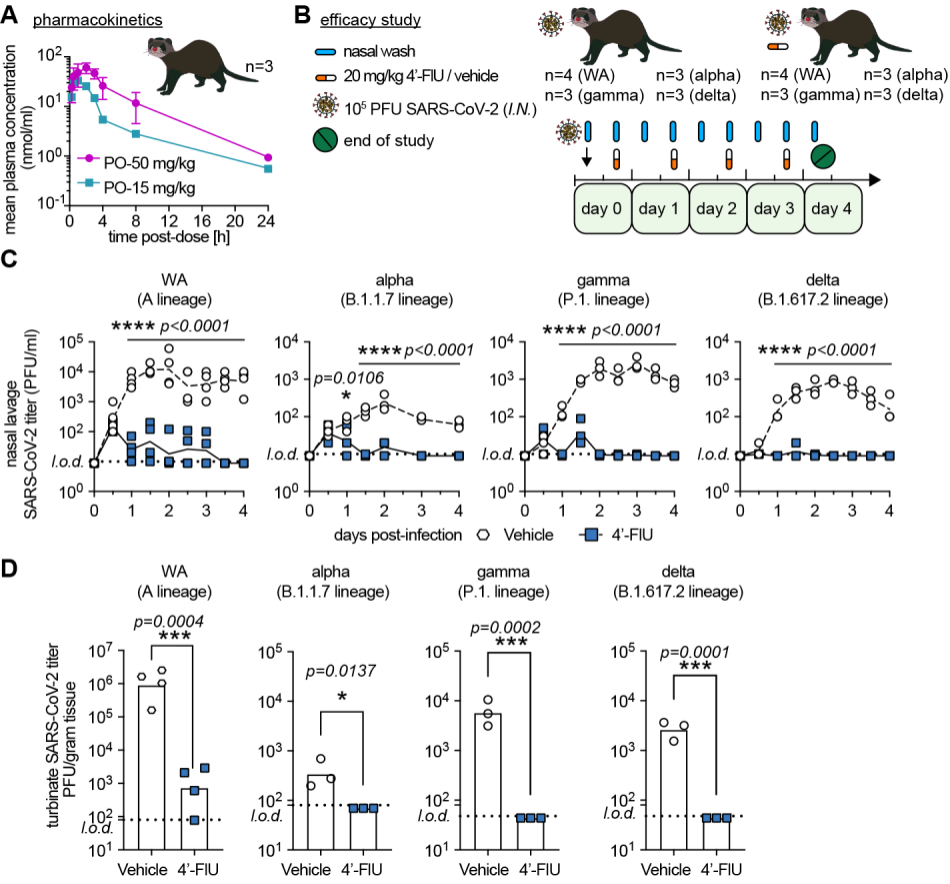
Therapeutic oral efficacy of 4′-FlU against different SARS-CoV-2 isolates in ferrets. (**A**) Single oral dose (15 or 50 mg/kg bodyweight) pharmacokinetics properties of 4′-FlU in ferret plasma (*n *= 3). (**B**) Ferrets were inoculated with SARS-CoV-2 WA1 or VoC alpha, gamma, or delta, and treated as indicated. (**C**) Nasal lavages were performed twice daily and viral titers were determined by plaque assay [*n *= 4 (WA1) or *n *= 3 (alpha, gamma, delta)]. (**D**) Viral titers in nasal turbinates at day 4 post-infection. In all panels, symbols represent individual independent biological repeats and lines show mean values. Two-way ANOVA with Sidak’s post hoc multiple comparison (C) and unpaired *t*-test (D).

To probe for a corresponding antiviral effect in vivo, we determined efficacy of oral 4′-FlU against an early pandemic isolate (WA1) and VoC alpha, gamma, and delta in the ferret model (*27*), which recapitulates hallmarks of uncomplicated human infection (*3*). For dose level selection in ferrets, we determined single oral dose ferret pharmacokinetic (PK) profiles of 4′-FlU. When administered at 15 or 50 mg/kg, peak plasma concentrations (C_max_) of 4′-FlU reached 34.8 and 63.3 μM, respectively, and overall exposure was 154 ± 27.6 and 413.1 ± 78.1 hours×nmol/ml, respectively, revealing good oral dose-proportionality (Fig. 6A and table S3). On the basis of this PK performance, we selected once-daily dosing at 20 mg/kg body weight for efficacy tests (Fig. 6B).

Intranasal infection of ferrets with 1×10^5^ PFU of each isolate resulted in rapid viral shedding into the upper respiratory tract, which plateaued in vehicle-treated animals 48 to 60 hours after infection (Fig. 6C). Therapeutic treatment with 4′-FlU initiated 12 hours after infection reduced virus burden in nasal lavages by approximately three orders of magnitude (WA1) to <50 PFU/ml within 12 hours of treatment onset. All three VoC were highly sensitive to 4′-FlU, remaining below the level of detection 36 to 48 hours after onset of oral treatment. Viral titers in nasal turbinate tissue extracted 4 days after infection (Fig. 6D) and associated viral RNA copy numbers (fig. S16) correlated with this reduction in shed virus load. Shedding of infectious particles ceased completely in all animals after 2.5 days of treatment (3 days post-infection).

## Conclusions

This study identifies and characterizes the ribonucleoside analog 4′-FlU, which potently inhibits pathogens of different clinically-relevant negative and positive-sense RNA virus families. The compound causes delayed stalling of RSV and SARS-CoV-2 polymerases within in vitro RdRP assays, reminiscent of the antiviral effect of remdesivir (*28*, *29*). However, 4′-FlU can also trigger immediate RdRP stalling depending on sequence context, suggesting steric hindrance of polymerase advance or of accommodating the next incoming nucleotide as the underlying MOA. We cannot exclude that additional effects further enhance the antiviral effect in cellula as proposed for other nucleoside analogs (*30*). Slightly lower sensitivity of SARS-CoV-2 to 4′-FlU compared with RSV could be a result of the exonuclease activity of the coronavirus polymerase, which can eliminate ribonucleoside analogs (*31*, *32*). Alternatively, coronavirus RdRP may have a greater capacity to tolerate the compound, because SARS-CoV-2 RdRP showed a higher tendency than RSV polymerase to advance after 4′-FlU-TP incorporation in the RdRP assays, which do not contain exonuclease functionality.

Once-daily oral administration to mice and ferrets significantly reduced the burden of RSV and SARS-CoV-2, respectively, when treatment was initiated up to 24 (RSV) or 12 (SARS-CoV-2) hours after infection. Because RSV (*33*) and SARS-CoV-2 (*34*) host invasion is slower in humans, these data outline a viable therapeutic window for human treatment. Equally potent activity against SARS-CoV-2 VoC alpha, gamma, and delta demonstrated broad anti-coronavirus efficacy of 4′-FlU, building confidence that the compound will remain active against future VoC that may be increasingly less responsive to spike-targeting vaccines or antibody therapeutics. Formal tolerability studies are pending, but 4′-FlU was well tolerated by the human organoid models and efficacious in murids and mustelids. Blood analysis of treated mice uncovered no antiproliferative effect of 4′-FlU on the hematopoietic system. These results establish 4′-FlU as a broad-spectrum orally efficacious inhibitor of major RNA viruses, making it a promising therapeutic option for RSV disease and COVID-19, and a much-needed contributor to improving pandemic preparedness.

## Supplementary Materials

science.org/doi/10.1126/science.abj5508
Materials and Methods Supplementary Text Figs. S1 to S18 Tables S1 to S3 References (*35*, *36*) MDAR Reproducibility Checklist Data S1 and S2

## References

[1] J. H. Beigel, K. M. Tomashek, L. E. Dodd, A. K. Mehta, B. S. Zingman, A. C. Kalil, E. Hohmann, H. Y. Chu, A. Luetkemeyer, S. Kline, D. Lopez de Castilla, R. W. Finberg, K. Dierberg, V. Tapson, L. Hsieh, T. F. Patterson, R. Paredes, D. A. Sweeney, W. R. Short, G. Touloumi, D. C. Lye, N. Ohmagari, M. D. Oh, G. M. Ruiz-Palacios, T. Benfield, G. Fätkenheuer, M. G. Kortepeter, R. L. Atmar, C. B. Creech, J. Lundgren, A. G. Babiker, S. Pett, J. D. Neaton, T. H. Burgess, T. Bonnett, M. Green, M. Makowski, A. Osinusi, S. Nayak, H. C. Lane; ACTT-1 Study Group Members, Remdesivir for the Treatment of Covid-19 - Final Report. N. Engl. J. Med. 383, 1813–1826 (2020). 10.1056/NEJMoa2007764 32445440 PMC7262788

[2] M. Toots, J.-J. Yoon, R. M. Cox, M. Hart, Z. M. Sticher, N. Makhsous, R. Plesker, A. H. Barrena, P. G. Reddy, D. G. Mitchell, R. C. Shean, G. R. Bluemling, A. A. Kolykhalov, A. L. Greninger, M. G. Natchus, G. R. Painter, R. K. Plemper, Characterization of orally efficacious influenza drug with high resistance barrier in ferrets and human airway epithelia. Sci. Transl. Med. 11, eaax5866 (2019). 10.1126/scitranslmed.aax5866 31645453 PMC6848974

[3] R. M. Cox, J. D. Wolf, R. K. Plemper, Therapeutically administered ribonucleoside analogue MK-4482/EIDD-2801 blocks SARS-CoV-2 transmission in ferrets. Nat. Microbiol. 6, 11–18 (2021). 10.1038/s41564-020-00835-2 33273742 PMC7755744

[4] G. R. Painter, R. A. Bowen, G. R. Bluemling, J. DeBergh, V. Edpuganti, P. R. Gruddanti, D. B. Guthrie, M. Hager, D. L. Kuiper, M. A. Lockwood, D. G. Mitchell, M. G. Natchus, Z. M. Sticher, A. A. Kolykhalov, The prophylactic and therapeutic activity of a broadly active ribonucleoside analog in a murine model of intranasal venezuelan equine encephalitis virus infection. Antiviral Res. 171, 104597 (2019). 10.1016/j.antiviral.2019.104597 31494195

[5] T. P. Sheahan, A. C. Sims, S. Zhou, R. L. Graham, A. J. Pruijssers, M. L. Agostini, S. R. Leist, A. Schäfer, K. H. Dinnon 3rd, L. J. Stevens, J. D. Chappell, X. Lu, T. M. Hughes, A. S. George, C. S. Hill, S. A. Montgomery, A. J. Brown, G. R. Bluemling, M. G. Natchus, M. Saindane, A. A. Kolykhalov, G. Painter, J. Harcourt, A. Tamin, N. J. Thornburg, R. Swanstrom, M. R. Denison, R. S. Baric, An orally bioavailable broad-spectrum antiviral inhibits SARS-CoV-2 in human airway epithelial cell cultures and multiple coronaviruses in mice. Sci. Transl. Med. 12, eabb5883 (2020). 10.1126/scitranslmed.abb5883 32253226 PMC7164393

[6] A. R. Falsey, P. A. Hennessey, M. A. Formica, C. Cox, E. E. Walsh, Respiratory syncytial virus infection in elderly and high-risk adults. N. Engl. J. Med. 352, 1749–1759 (2005). 10.1056/NEJMoa043951 15858184

[7] B. Rha, A. T. Curns, J. Y. Lively, A. P. Campbell, J. A. Englund, J. A. Boom, P. H. Azimi, G. A. Weinberg, M. A. Staat, R. Selvarangan, N. B. Halasa, M. M. McNeal, E. J. Klein, C. J. Harrison, J. V. Williams, P. G. Szilagyi, M. N. Singer, L. C. Sahni, D. Figueroa-Downing, D. McDaniel, M. M. Prill, B. L. Whitaker, L. S. Stewart, J. E. Schuster, B. A. Pahud, G. Weddle, V. Avadhanula, F. M. Munoz, P. A. Piedra, D. C. Payne, G. Langley, S. I. Gerber, Respiratory Syncytial Virus-Associated Hospitalizations Among Young Children: 2015-2016. Pediatrics 146, e20193611 (2020). 10.1542/peds.2019-3611 32546583 PMC12874392

[8] T. Shi, A. Denouel, A. K. Tietjen, I. Campbell, E. Moran, X. Li, H. Campbell, C. Demont, B. O. Nyawanda, H. Y. Chu, S. K. Stoszek, A. Krishnan, P. Openshaw, A. R. Falsey, H. Nair, H. Nair, H. Campbell, T. Shi, S. Zhang, Y. Li, P. Openshaw, J. Wedzicha, A. Falsey, M. Miller, P. Beutels, L. Bont, A. Pollard, E. Molero, F. Martinon-Torres, T. Heikkinen, A. Meijer, T. K. Fischer, M. van den Berge, C. Giaquinto, R. Mikolajczyk, J. Hackett, B. Cai, C. Knirsch, A. Leach, S. K. Stoszek, S. Gallichan, A. Kieffer, C. Demont, A. Denouel, A. Cheret, S. Gavart, J. Aerssens, R. Fuentes, B. Rosen, H. Nair, H. Campbell, T. Shi, S. Zhang, Y. Li, P. Openshaw, J. Wedzicha, A. Falsey, M. Miller, P. Beutels, L. Bont, A. Pollard, E. Molero, F. Martinon-Torres, T. Heikkinen, A. Meijer, T. K. Fischer, M. van den Berge, C. Giaquinto, R. Mikolajczyk, J. Hackett, B. Cai, C. Knirsch, A. Leach, S. K. Stoszek, S. Gallichan, A. Kieffer, C. Demont, A. Denouel, A. Cheret, S. Gavart, J. Aerssens, R. Fuentes, B. Rosen; RESCEU Investigators, Global Disease Burden Estimates of Respiratory Syncytial Virus-Associated Acute Respiratory Infection in Older Adults in 2015: A Systematic Review and Meta-Analysis. J. Infect. Dis. 222 (Suppl 7), S577–S583 (2020). 10.1093/infdis/jiz059 30880339

[9] T. Shi, D. A. McAllister, K. L. O’Brien, E. A. F. Simoes, S. A. Madhi, B. D. Gessner, F. P. Polack, E. Balsells, S. Acacio, C. Aguayo, I. Alassani, A. Ali, M. Antonio, S. Awasthi, J. O. Awori, E. Azziz-Baumgartner, H. C. Baggett, V. L. Baillie, A. Balmaseda, A. Barahona, S. Basnet, Q. Bassat, W. Basualdo, G. Bigogo, L. Bont, R. F. Breiman, W. A. Brooks, S. Broor, N. Bruce, D. Bruden, P. Buchy, S. Campbell, P. Carosone-Link, M. Chadha, J. Chipeta, M. Chou, W. Clara, C. Cohen, E. de Cuellar, D.-A. Dang, B. Dash-Yandag, M. Deloria-Knoll, M. Dherani, T. Eap, B. E. Ebruke, M. Echavarria, C. C. de Freitas Lázaro Emediato, R. A. Fasce, D. R. Feikin, L. Feng, A. Gentile, A. Gordon, D. Goswami, S. Goyet, M. Groome, N. Halasa, S. Hirve, N. Homaira, S. R. C. Howie, J. Jara, I. Jroundi, C. B. Kartasasmita, N. Khuri-Bulos, K. L. Kotloff, A. Krishnan, R. Libster, O. Lopez, M. G. Lucero, F. Lucion, S. P. Lupisan, D. N. Marcone, J. P. McCracken, M. Mejia, J. C. Moisi, J. M. Montgomery, D. P. Moore, C. Moraleda, J. Moyes, P. Munywoki, K. Mutyara, M. P. Nicol, D. J. Nokes, P. Nymadawa, M. T. da Costa Oliveira, H. Oshitani, N. Pandey, G. Paranhos-Baccalà, L. N. Phillips, V. S. Picot, M. Rahman, M. Rakoto-Andrianarivelo, Z. A. Rasmussen, B. A. Rath, A. Robinson, C. Romero, G. Russomando, V. Salimi, P. Sawatwong, N. Scheltema, B. Schweiger, J. A. G. Scott, P. Seidenberg, K. Shen, R. Singleton, V. Sotomayor, T. A. Strand, A. Sutanto, M. Sylla, M. D. Tapia, S. Thamthitiwat, E. D. Thomas, R. Tokarz, C. Turner, M. Venter, S. Waicharoen, J. Wang, W. Watthanaworawit, L.-M. Yoshida, H. Yu, H. J. Zar, H. Campbell, H. Nair; RSV Global Epidemiology Network, Global, regional, and national disease burden estimates of acute lower respiratory infections due to respiratory syncytial virus in young children in 2015: A systematic review and modelling study. Lancet 390, 946–958 (2017). 10.1016/S0140-6736(17)30938-8 28689664 PMC5592248

[10] G. S. Cockerill, J. A. D. Good, N. Mathews, State of the Art in Respiratory Syncytial Virus Drug Discovery and Development. J. Med. Chem. 62, 3206–3227 (2019). 10.1021/acs.jmedchem.8b01361 30411898

[11] R. Fearns, R. K. Plemper, Polymerases of paramyxoviruses and pneumoviruses. Virus Res. 234, 87–102 (2017). 10.1016/j.virusres.2017.01.008 28104450 PMC5476513

[12] R. M. Cox, M. Toots, J.-J. Yoon, J. Sourimant, B. Ludeke, R. Fearns, E. Bourque, J. Patti, E. Lee, J. Vernachio, R. K. Plemper, Development of an allosteric inhibitor class blocking RNA elongation by the respiratory syncytial virus polymerase complex. J. Biol. Chem. 293, 16761–16777 (2018). 10.1074/jbc.RA118.004862 30206124 PMC6204889

[13] J. DeVincenzo, L. Cass, A. Murray, K. Woodward, E. Meals, M. Coates, L. Daly, V. Wheeler, J. Mori, C. Brindley, A. Davis, M. McCurdy, K. Ito, B. Murray, P. Strong, G. Rapeport, Safety and Anti-viral Effects of Nebulized PC786 in a Respiratory Syncytial Virus Challenge Study. J. Infect. Dis. jiaa716 (2020). 10.1093/infdis/jiaa716 33216113 PMC9200148

[14] J. J. Yoon, M. Toots, S. Lee, M.-E. Lee, B. Ludeke, J. M. Luczo, K. Ganti, R. M. Cox, Z. M. Sticher, V. Edpuganti, D. G. Mitchell, M. A. Lockwood, A. A. Kolykhalov, A. L. Greninger, M. L. Moore, G. R. Painter, A. C. Lowen, S. M. Tompkins, R. Fearns, M. G. Natchus, R. K. Plemper, Orally Efficacious Broad-Spectrum Ribonucleoside Analog Inhibitor of Influenza and Respiratory Syncytial Viruses. Antimicrob. Agents Chemother. 62, ••• (2018). 10.1128/AAC.00766-18 29891600 PMC6105843

[15] P. Richardson, Applications of fluorine to the construction of bioisosteric elements for the purposes of novel drug discovery. Expert Opin. Drug Discov. 16, 1261–1286 (2021). 10.1080/17460441.2021.1933427 34074189

[16] M. L. Moore, M. H. Chi, C. Luongo, N. W. Lukacs, V. V. Polosukhin, M. M. Huckabee, D. C. Newcomb, U. J. Buchholz, J. E. Crowe Jr., K. Goleniewska, J. V. Williams, P. L. Collins, R. S. Peebles Jr., A chimeric A2 strain of respiratory syncytial virus (RSV) with the fusion protein of RSV strain line 19 exhibits enhanced viral load, mucus, and airway dysfunction. J. Virol. 83, 4185–4194 (2009). 10.1128/JVI.01853-08 19211758 PMC2668460

[17] L. D. Marroquin, J. Hynes, J. A. Dykens, J. D. Jamieson, Y. Will, Circumventing the Crabtree effect: Replacing media glucose with galactose increases susceptibility of HepG2 cells to mitochondrial toxicants. Toxicol. Sci. 97, 539–547 (2007). 10.1093/toxsci/kfm052 17361016

[18] S. P. Luby, The pandemic potential of Nipah virus. Antiviral Res. 100, 38–43 (2013). 10.1016/j.antiviral.2013.07.011 23911335

[R19] 19Note: Based on the current knowledge of the pyrimidine salvage pathway, it must be assumed that the large excess of extracellular cytidine leads to conversion of CTP to UTP via cytidine deaminase (CDA), competing with 4′-FlU.

[20] B. Ludeke, R. Fearns, The respiratory syncytial virus polymerase can perform RNA synthesis with modified primers and nucleotide analogs. Virology 540, 66–74 (2020). 10.1016/j.virol.2019.11.002 31739186 PMC7737601

[21] E. P. Tchesnokov, J. Y. Feng, D. P. Porter, M. Götte, Mechanism of Inhibition of Ebola Virus RNA-Dependent RNA Polymerase by Remdesivir. Viruses 11, 326 (2019). 10.3390/v11040326 30987343 PMC6520719

[22] C. J. Gordon, E. P. Tchesnokov, E. Woolner, J. K. Perry, J. Y. Feng, D. P. Porter, M. Götte, Remdesivir is a direct-acting antiviral that inhibits RNA-dependent RNA polymerase from severe acute respiratory syndrome coronavirus 2 with high potency. J. Biol. Chem. 295, 6785–6797 (2020). 10.1074/jbc.RA120.013679 32284326 PMC7242698

[23] T. L. Dangerfield, N. Z. Huang, K. A. Johnson, Expression and purification of tag-free SARS-CoV-2 RNA-dependent RNA polymerase in *Escherichia coli*. STAR Protoc 2, 100357 (2021). 10.1016/j.xpro.2021.100357 33558863 PMC7859716

[R24] 24Note: Our nsp12 preparations contain a mixture of ‘GroEL’ and ‘tf’, and nsp12 as described before, the latter representing 19% and 18% of the protein content of the preparations containing catalytically active and inactive nsp12, respectively.

[25] M. Toots, J.-J. Yoon, M. Hart, M. G. Natchus, G. R. Painter, R. K. Plemper, Quantitative efficacy paradigms of the influenza clinical drug candidate EIDD-2801 in the ferret model. Transl. Res. 218, 16–28 (2020). 10.1016/j.trsl.2019.12.002 31945316 PMC7568909

[26] S. M. Coleman, A. McGregor, A bright future for bioluminescent imaging in viral research. Future Virol. 10, 169–183 (2015). 10.2217/fvl.14.96 26413138 PMC4581531

[R27] 27Note: Male ferrets were not included in this study for practical reasons as they are territorial and available ABSL3 space cannot accommodate the number of cages that this study would require to achieve meaningful statistical power.

[28] G. Kokic, H. S. Hillen, D. Tegunov, C. Dienemann, F. Seitz, J. Schmitzova, L. Farnung, A. Siewert, C. Höbartner, P. Cramer, Mechanism of SARS-CoV-2 polymerase stalling by remdesivir. Nat. Commun. 12, 279 (2021). 10.1038/s41467-020-20542-0 33436624 PMC7804290

[29] E. P. Tchesnokov, C. J. Gordon, E. Woolner, D. Kocinkova, J. K. Perry, J. Y. Feng, D. P. Porter, M. Götte, Template-dependent inhibition of coronavirus RNA-dependent RNA polymerase by remdesivir reveals a second mechanism of action. J. Biol. Chem. 295, 16156–16165 (2020). 10.1074/jbc.AC120.015720 32967965 PMC7681019

[30] J. J. Feld, J. H. Hoofnagle, Mechanism of action of interferon and ribavirin in treatment of hepatitis C. Nature 436, 967–972 (2005). 10.1038/nature04082 16107837

[31] E. C. Smith, H. Blanc, M. C. Surdel, M. Vignuzzi, M. R. Denison, Coronaviruses lacking exoribonuclease activity are susceptible to lethal mutagenesis: Evidence for proofreading and potential therapeutics. PLOS Pathog. 9, e1003565 (2013). 10.1371/journal.ppat.1003565 23966862 PMC3744431

[32] E. Minskaia, T. Hertzig, A. E. Gorbalenya, V. Campanacci, C. Cambillau, B. Canard, J. Ziebuhr, Discovery of an RNA virus 3′->5′ exoribonuclease that is critically involved in coronavirus RNA synthesis. Proc. Natl. Acad. Sci. U.S.A. 103, 5108–5113 (2006). 10.1073/pnas.0508200103 16549795 PMC1458802

[33] C. M. El Saleeby, A. J. Bush, L. M. Harrison, J. A. Aitken, J. P. Devincenzo, Respiratory syncytial virus load, viral dynamics, and disease severity in previously healthy naturally infected children. J. Infect. Dis. 204, 996–1002 (2011). 10.1093/infdis/jir494 21881113 PMC3203391

[34] B. Oberfeld, A. Achanta, K. Carpenter, P. Chen, N. M. Gilette, P. Langat, J. T. Said, A. E. Schiff, A. S. Zhou, A. K. Barczak, S. Pillai, SnapShot: COVID-19. Cell 181, 954–954.e1 (2020). 10.1016/j.cell.2020.04.013 32413300 PMC7190493

[35] C. Ye, K. Chiem, J.-G. Park, J. A. Silvas, D. Morales Vasquez, J. Sourimant, M. J. Lin, A. L. Greninger, R. K. Plemper, J. B. Torrelles, J. J. Kobie, M. R. Walter, J. C. de la Torre, L. Martinez-Sobrido, Analysis of SARS-CoV-2 infection dynamic in vivo using reporter-expressing viruses. Proc. Natl. Acad. Sci. U.S.A. 118, e2111593118 (2021). 10.1073/pnas.2111593118 34561300 PMC8521683

[36] M. A. Rameix-Welti, R. Le Goffic, P.-L. Hervé, J. Sourimant, A. Rémot, S. Riffault, Q. Yu, M. Galloux, E. Gault, J.-F. Eléouët, Visualizing the replication of respiratory syncytial virus in cells and in living mice. Nat. Commun. 5, 5104 (2014). 10.1038/ncomms6104 25277263 PMC7091779

